# Laparoscopic appendicectomy is a favorable alternative for complicated appendicitis in children

**DOI:** 10.4103/0971-9261.43797

**Published:** 2008

**Authors:** J. Deepak, Prakash Agarwal, R. K. Bagdi, S. Balagopal, R. Madhu, P. Balamourougane, Zaffer Saleem Khanday

**Affiliations:** Department of Pediatric Surgery, Sri Ramachandra Medical College and Research Institute, Chennai, India

**Keywords:** Complicated appendicitis, laparoscopic appendicectomy, open appendicectomy

## Abstract

**Aim:**

To evaluate the role of laparoscopy in complicated appendicitis in children.

**Materials and Methods:**

A total of 119 children were operated for appendicitis between October 2005 and May 2008 at SRMC, Chennai. Forty-one patients underwent open appendicectomy (OA), and 71 patients underwent laparoscopic appendicectomy (LA). Twenty-six cases among the LA group and 16 among the OA group had complicated appendicitis. Twenty-six cases were completed laparoscopically, and 2 needed conversion to OA.

**Results:**

Out of 26 patients in the LA group, 23 made an uneventful recovery without any complications. One had minor port site infection, and 2 had prolonged loose stools. Out of 16 in the OA group, 7 had complications. Three had wound infection, 2 had loose stools, 1 had fecal fistula and another required subsequent surgeries. Operative duration in LA was 86.7 min (range: 75 to 120 min) and 90.3 min (range: 70 to 150 min) in OA. Oral feed resumption in LA was done at average of 2.7 days and in OA at 4.3 days. IV antibiotics were administered for an average of 3.6 days in LA and 4.8 days in OA, parenteral analgesic for 2.7 days in LA and 4.2 days in OA. The length of hospital stay was 5.4 days in LA and 7.3 days in OA.

**Conclusion:**

LA is a favorable alternative in children with complicated appendicitis in view of less postoperative pain, fewer postoperative complications and quicker return to normal activity.

Complicated appendicitis (CA) is a common surgical emergency in childhood, more so in developing countries with poor patient education and limited access to hospital with advanced surgical facilities. In the era of minimal access surgery, there is still a controversy regarding the modality of treatment for CA, whether open appendicectomy (OA) or laparoscopic appendectomy (LA) should be done. In many centers across the world, LA has been a routine for simple appendicitis in children. However, the role of laparoscopic approach for CA in children is still debatable. We have done a hospital-based study for LA in comparison with OA for children with CA in our institution and analyzed the feasibility, safety and benefits of LA.

## MATERIALS AND METHODS

A total of 119 children with preoperative diagnosis of acute or recurrent appendicitis were operated upon between October 2005 and May 2008 in our institution. Seventy-eight children underwent LA and 41 underwent OA. 26 patients in the LA group and 16 patients in OA group had complicated appendicitis (CA). In this study, we analysed the results of 42 children with CA. CA was defined as acute or recurrent appendicitis associated with gangrene, appendicular mass, perforation of appendix or abscess formation with localized or generalized peritonitis. The diagnosis was confirmed intra-operatively and on histopathology. The segregation of patients was according to the procedure performed, either LA or OA groups. OA and LA were performed by different pediatric surgeons. The choice of procedure was dependent on the surgeon’s preference. Patient demographics, operative findings, duration of surgery and operative techniques were recorded. The details of the postoperative outcome in terms of resumption of oral feeds, duration of need for parenteral analgesia, duration of intravenous antibiotics, length of hospitalization and post-operative complications were analyzed.

**Figure 1:**
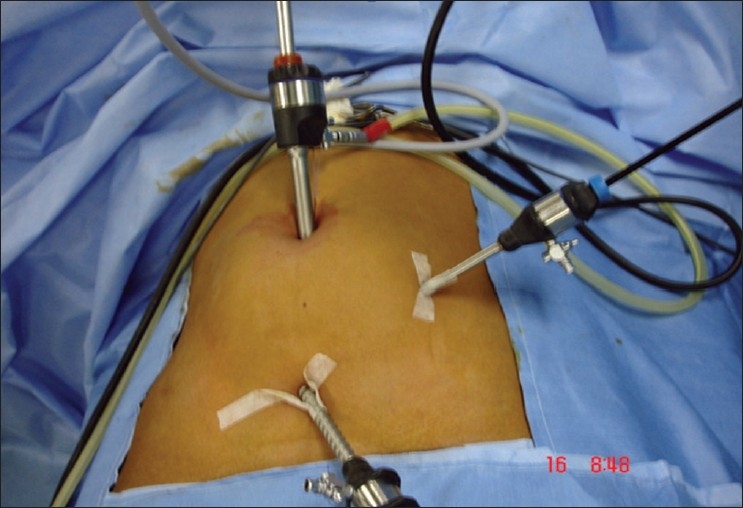
Diagram showing sites of port insertion for laparoscopic appendicectomy

Laparoscopic appendectomy (LA) was performed through 3 ports [Figure 1]. A 10-mm infraumblical camera port inserted by open technique. Pneumoperitoneum to a pressure of 10-14 mm Hg was achieved by carbon dioxide insufflation. Two 5-mm working ports were inserted under vision in the left iliac fossa and suprapubic region. The appendix was dissected out and the mesoappendix cauterized using monopolar diathermy attached to either a hook or grasping forceps. The appendicular base was ligated with pre-tied chromic catgut or polyglactin endoloop and divided. The appendix was retrieved through 10-mm umbilical port. The small bowel was traced from the ileocecal junction, proximally up to duodenojejunal flexure with atraumatic grasper in all cases. Interloop adhesions were released and pus cavities drained when encountered. In the case of peritonitis, abdominal irrigation with normal saline was performed until the aspirated fluid became clear. Closed tube drains were placed when deemed necessary.

Open appendicectomy (OA) was performed through right lower quadrant Lanz incision extending it on either side or cutting the muscle along the incision when required. Pus pockets drained and peritoneal lavage was given in cases of peritonitis. Corrugated, intraperitoneal drain was placed when required.

Postoperatively, intravenous cefotaxime or ceftriaxone, amikacin and metronidazole were administered until culture reports were obtained and patient tolerated a normal oral diet. Later, oral cefixime was administered for 5 days in all the patients after stopping iv antibiotics. Rectal paracetamol or diclofenac suppository was given for analgesia in all the patients. Oral feeds were started with resumption of bowel activity. Nasogastric tube was used routinely in all patients. All appendices were examined histologically. Pus was sent for culture and drug sensitivity. The patients were discharged when they were able to tolerate regular diet and were ambulatory. They were followed up in the outpatient clinic at least once after their discharge from the hospital. Results were analyzed using the T-test and Chi-square test.

## RESULTS

Forty-two children (23 males and 19 females) with a mean age of 10.70 years (range: 1 month to 17 years) presented with complicated appendicitis. Twenty-six children underwent LA (15 males and 11 females), and 16 children (8 males and 8 females) underwent OA. Both groups were comparable in terms of patient demographics, duration of symptoms and operative findings [Table 1].

**Table 1:** Comparison of demographic data, symptoms and operative findings between laparoscopic and open appendicectomy groups

	Laparoscopic appendicectomy group (n = 26)	Open appendicectomy group (n = 16)
Sex (Male:Female)	15:11	8:8
Age in years [mean, (range)]	10.1 (5 to 17)	11.5 (1 month to 17 years)
Pain duration in days (range)	2.7 (1 to 10)	2.9 (1 to 10)
Operative Findings (percentage)		
Appendiceal perforation	14 (57.6%)	7 (43.8%)
Appendiceal mass	8 (31.8%)	6 (37.5%)
Appendiceal abscess	1 (3.8%)	1 (6.2%)
Appendiceal gangrene	2 (7.7%)	1 (6.2%)
Appendicitis with band causing intestinal obstruction	0 (0 %)	1 (6.2%)

Raised total leukocyte count (above 14,000/cu mm) was found in 35.7% of our patients only. However, the percentages of polymorphs were uniformly increased (constituting more than 70% of total count) in all the 42 patients. The operative duration was comparable in LA (mean: 86.7 min) and OA (mean: 90.3 min) group. Operative findings in 26 patients belonging to LA group include appendicular perforation in 15 (57.7%), appendicular mass in 8 (31.8%), appendicular abscess in 1 (3.8%) and gangrenous appendix in 2 (7.7%). Among 16 patients of the OA group, 7 (43.8%) had appendicular perforation, 6 (37.5%) had appendicular mass, 1 each had appendicular abscess, gangrenous appendix and appendicitis with fibrinous band causing small intestinal obstruction. The last case was a 40-day-old infant who presented with features suggestive of acute intestinal obstruction. Fecoliths were found in 7 (26.9%) cases in LA and 4 (25%) cases in OA group.

Intraperitoneal drains were placed in 19 (73.1%) patients in the LA group and 09 (56.2%) patients in the OA group. The mean duration of drain placement postoperatively was 3.4 days for LA and 5.9 days for OA. The duration of intravenous antibiotics used postoperatively was 3.5 days for LA and 4.8 days for OA (*P* = 0.032). Rectal analgesic suppositories were required for 2.7 days in LA and 4.2 days in OA (*P* = 0.007). Postoperatively, time required for resumption of full oral feeds was 2.7 days for LA and 4.3 days for OA (*P* = 0.004). The mean length of hospital stay was shorter in the LA (5.4 days) than that in the OA group (7.3 days) (*P* = 0.057). The above data is summarized in Table 2.

**Table 2:** Comparison of operative duration and postoperative course between laparoscopic and open appendicectomy groups

	Laparoscopic appendicectomy (n = 26) [mean (range)]	Open appendicectomy (n = 16) [mean (range)]	*P*
Operative duration (min)	86.7 (75 to 120)	90.3 (70 to 150)	0.465
Oral feed resumption (days)	2.7 (2 to 5)	4.3 (3 to 12)	0.004
Intravenous antibiotic duration (days)	3.6 (2 to 6)	4.8 (3 to 12)	0.032
Parenteral analgesic duration (days)	2.7 (2 to 5)	4.2 (3 to 12)	0.007
Length of hospital stay (days)	5.4 (3 to 11)	7.3 (4 to 19)	0.057

Postoperative complications include superficial wound infection, postoperative loose stools, fecal fistula and requirement of a subsequent surgery [Table 3]. The incidence of these complications was 11.5% in the LA group and 43.8% in the OA group (*P* = 0.027). In the LA group, 1 patient (3.8%) had superficial umbilical port site wound infection, whereas in the OA group, 3 patients (18.7%) had superficial wound infection. Post-operative loose stools were present for more than 2 days in 2 patients (7.7%) in the LA group and 2 patients (12.5%) in the OA group. One case which was converted from laparoscopy to open procedure due to extensive pus with fecal peritonitis had sloughed off appendix with friable base, who later developed fecal fistula on post-operative day (POD) 8, which resolved completely by conservative management. Another patient in the OA group had an appendicular abscess with small bowel serositis and dusky discoloration of sigmoid colon which was exteriorized. Since that part of sigmoid was not viable, excision of the exteriorized segment of sigmoid colon and colostomy maturation was done on POD 3; subsequent colostomy closure was done 6 weeks later. There was no mortality in either group. The mean duration of follow up was 12.8 months (range: 1–30 months). Two cases that were started laparoscopically and converted to open appendicectomy are included in the OA group for the study. The first patient was a 13–year-old female in whom pus was encountered as the umbilical port was inserted and we decided to convert it, in view of extensive intraperitoneal pus with fecal peritonitis. Second patient was an 8-year-old female in whom an appendicular mass with extensive inter loop adhesion was found and was converted into an open procedure.

**Table 3:** Comparison of postoperative complications between laparoscopic and open appendicectomy groups

	Laparoscopic appendicectomy Group (n = 3) [11.5%]	Open appendicectomy group (n = 7) [43.8%]	*P* = 0.017
Superficial wound infection [n (%)]	1 (3.8%)	3 (18.7%)	
Loose stools [n (%)]	2 (7.7%)	2 (12.5%)	
Fecal fistula [n (%)]	0	1 (6.25%)	
Subsequent surgery requirement [n (%)]	0	1 (6.25%)	

## DISCUSSION

LA has become routine for simple appendicitis in children in many centers across the world. However, the role of laparoscopic approach for CA in children is still debatable.[1–15] LA in CA has been reported to offer increased safety, shorter length of hospital stay, less pain and quicker return to normal activity with fewer complications.[1–5] In contrast, it has also been reported that LA in CA is associated with higher risks of postoperative intra-abdominal abscess formation, bleeding and bowel injuries.[6–8] Increased post-operative complications following conversion from LA to OA have also been reported.[9]

Our results have indicated the feasibility, safety and efficacy of LA in CA. The benefits of LA in CA were more obvious in the postoperative recovery. The duration of intravenous antibiotics, postoperative resumption of oral feeds and parenteral analgesics requirement were significantly shorter in the LA group when compared to the OA group. The operative time was comparable in both LA and OA groups. The length of hospitalization was shorter in the LA group, which is related to less pain, quicker ambulation, early resumption of oral feeds and fewer complications. This is attributed to less access trauma; the muscle cutting incision in OA is more painful and takes longer time to heal compared to muscle stretching port insertion. The complications encountered in the LA group were significantly less and minor. Postoperative fecal fistula, that occurred in the converted case due to leakage from the friable appendicular stump, inspite of a transfixing ligature of the stump, was more related to pathological condition of the patient, and was possibly unavoidable. Postoperative loose stools were treated with oral antibiotics and probiotics for 5 to 7 days with eventual cure in all the patients. In our study, we did not encounter any case of postoperative intra-abdominal abscess or intestinal obstruction in either group. Superficial wound infection, which was present in 2 patients each of the LA and OA groups, was treated by regular dressing and oral antibiotics as outpatients.

In the hands of an experienced laparoscopic surgeon, LA provides the advantages of panoramic view with increased magnification, ability to visualize the hidden corners and clearance of purulent material as compared to the open technique. It has a steep learning curve, and the results tend to be better once the surgeon acquires necessary experience. In our study, both the conversions were done on the initial 2 cases; later, 24 consecutive cases were operated laparoscopically without any conversion or major complication. In open surgery, atypical localization of the appendix may require an extended or second incision. Laparoscopy avoids this, and it gives an aesthetically acceptable scar and less operative trauma.

Our study has inherent limitations of lack of randomization, shorter follow up and possible observer bias. Nevertheless, our results indicate that LA is safe, effective and beneficial in children with CA. In the presence of relevant expertise, we recommend LA as a favorable alternative for complicated appendicitis in children.
